# Christianity and the Sick

**Published:** 1888-06-02

**Authors:** 


### CHRISTIANITY AND THE SICK.

For many hundreds of years, ever since the beginning of the Christian Era, religion has been publicly concerned with the mitigation of sorrow and suffering. In the early history of these islands, when civilisation, as we understand it, had not yet laid her soft injunctions upon our ancestors, and even their virtues were of a severe and barbaric order, we find it was the religious bodies alone which recognised a duty to the sick and gave them shelter and tendance. We read that, neglected by all others, these offices were performed with almost superstitious reverence by the monks and friars, and just as in our time the State surrenders the care of the sick poor to the benevolence of private citizens, so in those far-off days the task was left to those upon whom no obligation rested but such as was comprised in the mandates and promptings of a compassionate creed.

This combination of philanthropy with religious sentiment has proved of the highest importance to both, and if in more recent times the conjunction has not been so palpable as in those days when a part of the revenues of the Church was systematically set apart for the support of charitable institutions, it is yet, we believe, recognised by most thoughtful people that in the religious instincts of the country lies the best promise of its practical benevolence.

The recurrence of Hospital Sunday affords the churches a welcome opportunity for active co-operation in the great work carried on by the hospitals of the metropolis. It would be difficult to exaggerate the value and importance of the imperial labours they perform. The voluntary hospitals of London provide nearly ten thousand beds, and minister to hundreds of thousands of out-patients. They do this at a yearly cost of about £650,000, of which one-half is provided by endowments—the gifts of past generations.

They deal with so huge a mass of suffering that its statistics are appalling, and not only do they tenderly minister to the sick and dying poor of this vast and ever-growing population, but from them goes forth that light of knowledge without which the rich man's home would be left dark and hopeless in the hour of sickness.

Such, then, are the institutions for which the churches are about to plead. They represent by far the greatest philanthropic endeavours of the age, and in a world instinct with suffering the limits of their usefulness are only bounded by their means. Yet probably no other undertakings of benevolence pursue their calling under circumstances so discouraging and debilitating. The hospitals of the greatest and richest City of the world starve amid plenty, and languish for want of an infinitesimal portion of the money expended annually in luxury and pleasure. The task of maintenance grows harder with every succeeding year, and the histories of nearly all our hospitals appear to be but records of never-ceasing and desperate efforts to keep open their doors.

It is a fact to be lamented, yet which cannot be too often insisted upon, that our hospitals are left dependent upon the alms of a small part of the community, and that the vast remainder, made up of persons of all classes, take no heed of their wants. If only a sense of the public obligation to hospitals could be aroused, these beneficent institutions would no longer be forced to labour under the harassing and enfeebling conditions inseparable from a perennial poverty. They would gain a giant's strength wherewith to do the giant's work expected of them.

The neglect of the public obligation to the hospitals is a theme worthy of the Church's warmest solicitude. No class of the community can be better acquainted than the ministers of religion with the value of these institutions, for none perhaps is so directly brought into contact with them. It is from the pulpit that the appeal in aid of the hospitals can be most forcibly directed, as it is from the rectory, the vicarage, and the manse that their help is so often invoked. The history of the Hospital Sunday Fund shows many a glowing example of sympathy and enthusiasm in a noble cause, and of grand results achieved by earnest workers. Who can doubt what would follow, if from every pulpit in this vast City issued on Hospital Sunday such advocacy as men give to a cause which has won their souls? Hospitals are the children of Christianity. What greater claim can they prefer upon Christian ministers and Christian congregations? "What man is there of you whom if his son ask bread, will he give him a stone?"

